# The Oncological Outcome of Postoperative Radiotherapy in Patients with Node-Negative Early-Stage (T1/T2/N0) Oral Squamous Cell Carcinoma and Perineural Invasion: A Meta-Analysis

**DOI:** 10.3390/cancers17050862

**Published:** 2025-03-03

**Authors:** Karthik N. Rao, M. P. Sreeram, Remco de Bree, William M. Mendenhall, Primož Strojan, Göran Stenman, Antti Mäkitie, Alfons Nadal, Juan P. Rodrigo, Sweet Ping Ng, June Corry, Alessandra Rinaldo, Avraham Eisbruch, Alfio Ferlito

**Affiliations:** 1 Department of Head and Neck Oncology, Sri Shankara Cancer Foundation, Bangalore 560004, India; sreeram.mp@sschrc.org; 2 Department of Head and Neck Surgical Oncology, Division of Imaging and Oncology Center, University Medical Center Utrecht, Heidelberglaan 100, 3584 CX Utrecht, The Netherlands; r.debree@umcutrecht.nl; 3 Department of Radiation Oncology, University of Florida College of Medicine, Gainesville, FL 32610, USA; mendwm@shands.ufl.edu; 4 Department of Radiation Oncology, Institute of Oncology Ljubljana, Faculty of Medicine, University of Ljubljana, 1000 Ljubljana, Slovenia; pstrojan@onko-i.si; 5 Department of Pathology, Sahlgrenska Center for Cancer Research, University of Gothenburg, Sahlgrenska University Hospital, 41345 Gothenburg, Sweden; goran.stenman@gu.se; 6 Research Program in Systems Oncology, Department of Otorhinolaryngology-Head and Neck Surgery, University of Helsinki and Helsinki University Hospital, FI-00029 HUS Helsinki, Finland; antti.makitie@hus.fi; 7 Department of Pathology, Hospital Clinic, Barcelona, Department of Basic Clinical Practice, School of Medicine, Universitat de Barcelona, 08036 Barcelona, Spain; anadal@clinic.cat; 8 Department of Otolaryngology, Hospital Universitario Central de Asturias, Instituto Universitario de Oncología del Principado de Asturias, University of Oviedo, Centro de Investigación Biomédica en Red de Cáncer (CIBERONC), 33004 Oviedo, Spain; jprodrigo@uniovi.es; 9 Department of Radiation Oncology, Austin Health, The University of Melbourne, Melbourne, VIC 3084, Australia; sweetping.ng@austin.org.au; 10 Division of Radiation Oncology, GenesisCare Radiation Oncology, St. Vincent’s Hospital, Melbourne, VIC 3065, Australia; june.corry@genesiscare.com; 11 ENT Unit, Policlinico Città di Udine, 33100 Udine, Italy; dottalerinaldo@gmail.com; 12 Department of Radiation Oncology, University of Michigan, Ann Arbor, MI 48109, USA; eisbruch@med.umich.edu; 13 Coordinator of the International Head and Neck Scientific Group, 35100 Padua, Italy; profalfioferlito@gmail.com

**Keywords:** oral squamous cell carcinoma, early stage, N0 neck, adjuvant therapy, radiotherapy

## Abstract

**Objective:** To evaluate the impact of postoperative radiotherapy (PORT) on oncological outcomes in node-negative early-stage oral squamous cell carcinoma (OSCC) with perineural invasion (PNI). **Methods**: A systematic review and meta-analysis was conducted using the PubMed, EMBASE, and Scopus databases for the period from 2000 to 2024. Studies comparing PORT versus observation in pN0 early-stage OSCC with PNI were included. Oncological outcomes assessed included overall survival (OS), disease-free survival (DFS), and local control (LC). A random-effects model was used to calculate log odds ratios, and heterogeneity was assessed using tau^2^, chi^2^, and I^2^ statistics. **Results**: Seven retrospective studies comprising 522 patients (281 PORT, 241 no-PORT) were included. The 3-year overall survival (OS) was 86.3% in the PORT group compared to 71.1% in the no-PORT group (logOR = −1.03, *p* = 0.0012), while the 5-year OS was 88.1% versus 77.3% (logOR = −0.97, *p* = 0.0061). Disease-free survival (DFS) also favored PORT, with 3-year DFS at 86.3% versus 58.1% (logOR = −1.19, *p* < 0.001) and 5-year DFS at 86.3% versus 55% (logOR = −0.78, *p* = 0.003). Local control (LC) was higher in the PORT group, with 3-year LC rates of 89% compared to 72.2% in the no-PORT group (logOR = −1.13, *p* = 0.025). **Conclusions**: PORT significantly improves OS, DFS, and LC in node-negative early-stage OSCC with PNI as the sole adverse feature.

## Simple Summary

This study explores whether radiation therapy after surgery improves survival and reduces cancer recurrence in patients with early-stage oral cancer that has spread along nerves but has not reached the lymph nodes. Researchers reviewed seven studies involving 522 patients, comparing those who received radiation after surgery to those who did not. The results showed that radiation significantly improved survival. After three years, 86.3% of patients who had radiation were alive compared to 71.1% of those who did not. At five years, survival was 88.1% with radiation and 77.3% without it. Radiation also helped prevent cancer from returning, with disease-free survival rates of 86.3% versus 58.1% at three years and 86.3% versus 55% at five years. Additionally, local control, meaning the ability to prevent cancer from coming back in the same area, was better with radiation (89% vs. 72.2% at three years). These findings suggest that radiation therapy can be a valuable addition to surgery for these patients, improving long-term outcomes. This research provides important guidance for doctors in making treatment decisions and may help more patients live longer, healthier lives.

## 1. Introduction

Oral squamous cell carcinoma (OSCC) is a significant health concern and ranks among the most common cancers worldwide [1]. According to the latest GLOBOCAN data, approximately 370,000 new cases of OSCC are diagnosed yearly [2]. Early-stage cancers may be treated with surgery alone. Patients with a significant risk of a recurrence undergo postoperative radiotherapy (PORT) with or without chemotherapy in selected cases [1].

A considerable proportion of OSCC cases, about 30 to 40%, present at an early stage (T1/T2/N0) [3,4]. PORT is recommended in cases with close or positive surgical margins, advanced-stage disease, including node positivity, and adverse histological features, such as lympho-vascular invasion (LVE) and perineural invasion (PNI) [5,6]. PNI has been recognized as an important adverse histological feature, associated with neck node positivity, extra-nodal extension (ENE), advanced disease, locoregional recurrence (LRR), and distant metastasis (DM) [7]. Notably, recent studies have shown that PORT, even for those patients with PNI alone, can improve oncological outcomes [8,9,10,11].

Few studies have specifically evaluated PNI as the only risk factor in early-stage T1/T2/N0 OSCC. However, most of the studies did not assess PNI in isolation for PORT, instead combining various T categories or focusing on advanced disease [12,13,14,15,16,17,18]. The incidence of perineural invasion in early-stage OSCC ranges from 12 to 20% [13,14,16,19,20,21]. This leads to a decision-making dilemma regarding whether to provide PORT in such settings.

Hence, our meta-analysis is the first of its kind to evaluate oncological outcomes following adjuvant PORT (to tumor bed and neck) for patients with T1/T2/N0 with PNI, comparing these outcomes between those receiving adjuvant PORT and those without.

## 2. Methodology

This study was conducted in alignment with the PRISMA (Preferred Reporting Items for Systematic Reviews and Meta-Analyses) [22] and AMSTAR (Assessing the Methodological Quality of Systematic Reviews) [23] guidelines. To gather a comprehensive collection of the published medical literature, a systematic search of the PubMed, Embase, and Scopus databases was conducted, focusing on English-language publications from 2000 to 2024.

The search strategy utilized the terms such as “Oral cancer”, “T1”, “T2”, “N0”, “Oral cavity”, “Carcinoma”, “Cancer”, “Tongue”, “Buccal mucosa”, “Alveolus”, “Perineural invasion”, “Adjuvant therapy”, “RT”, “Radiation therapy”, and “Outcomes”. Boolean operators (NOT, AND, OR) were used to refine the search results. The last retrieval was performed on 9 July 2024.

Two investigators, KNR and SMP, independently screened the retrieved articles based on the type of article, title, and abstract. Eligible articles were then pooled, followed by a thorough full-text analysis and further assessment of references using a snowball search method. KNR and SMP selected the articles, and any disagreements on inclusion were resolved by the senior authors.

This study included treatment-naive patients with pathological node-negative early-stage OSCC who had histologically confirmed PNI and underwent curative ablative surgery. Only original research articles published in peer-reviewed journals were considered if they compared PORT (to the tumor bed and neck) with observation in PNI-positive cases and reported at least one oncological outcome, such as OS, DFS, DSS, or LC, along with Kaplan–Meier graphs. Studies were excluded if they involved non-human subjects, neoadjuvant chemotherapy or radiation, adjuvant chemoradiotherapy or brachytherapy, prior oncological treatment, recurrent or second primary tumors, or overlapping study populations. Additionally, non-original research articles, including reviews, meeting abstracts, case reports, editorial letters, and studies with incomplete or insufficient data, were excluded.

Data extraction was performed independently by KNR and SMP, recording study characteristics such as author, year of publication, country, sample size, type of study, level of evidence, risk of bias, number of PNI+ patients, number of PNI+ patients receiving PORT, number of PNI+ patients not receiving PORT, and 3-year and 5-year OS, DFS, DSS, and LC. To derive the outcomes from the presented Kaplan–Meier plots, we employed a method involving the measurement of data points on the x-axis, specifically at the 3-year and 5-year time points. This process was facilitated by digitally recalibrating the provided Kaplan–Meier graphs through utilization of the webplotdigitizer platform [24]. This approach allowed us to precisely extract the corresponding coordinates along both the x-axis (time points at 3 and 5 years) and the y-axis (survival probabilities or event rates). By digitally recalibrating the graphs, we ensured accurate data extraction, minimizing estimation errors and improving the reliability of the derived outcomes. A meta-analysis was conducted when the outcome was reported by more than two studies [25].

The level of evidence was assessed independently by KNR and SMP using the Oxford Centre for Evidence-Based Medicine (OCEBM) criteria [26]. Methodological quality was evaluated using the Newcastle–Ottawa Scale [27], with scores ranging from 0 to 9, and all of the included articles scored 6. Risk of bias was assessed using the ROBINS-I tool, which covers domains such as confounding, selection, classification of interventions, deviation from proposed treatments, missing data, measurement of outcomes, and selection of reported results [28].

Statistical analysis included calculating effect sizes for dichotomous variables using the number of incidents in both experimental and control groups, with the outcome measure being the log odds ratio [29]. The DerSimonian–Laird estimator and Wald-type tests were used for hypothesis testing with a 95% confidence interval [30]. The random-effects model was chosen to account for variations in patient populations, treatment protocols, and follow-up durations across retrospective studies, ensuring more generalizable estimates. Effect size visualization was carried out through a forest plot, ordered by the year of study, and a Labbe plot to assess interstudy heterogeneity [31]. A funnel plot was used to measure publication bias, and influence diagnostics were conducted using studentized residuals, Cook’s distance, and weights. Heterogeneity was assessed using the DerSimonian–Laird model to estimate between-study variance (tau^2^), the Cochran Q-test, and the Higgins I^2^ statistic [32]. Outliers were examined using studentized residuals, and Cook’s distances were used to identify influential studies [33]. Bias assessment was conducted using Egger’s regression test for funnel plot asymmetry and a mixed-effects meta-regression model. The Breg rank correlation test was also employed to assess publication bias [34]. The comprehensive meta-analysis was performed using the R Project for Statistical Computing v4.3.1 for Windows.

## 3. Results

**Figure 1:**
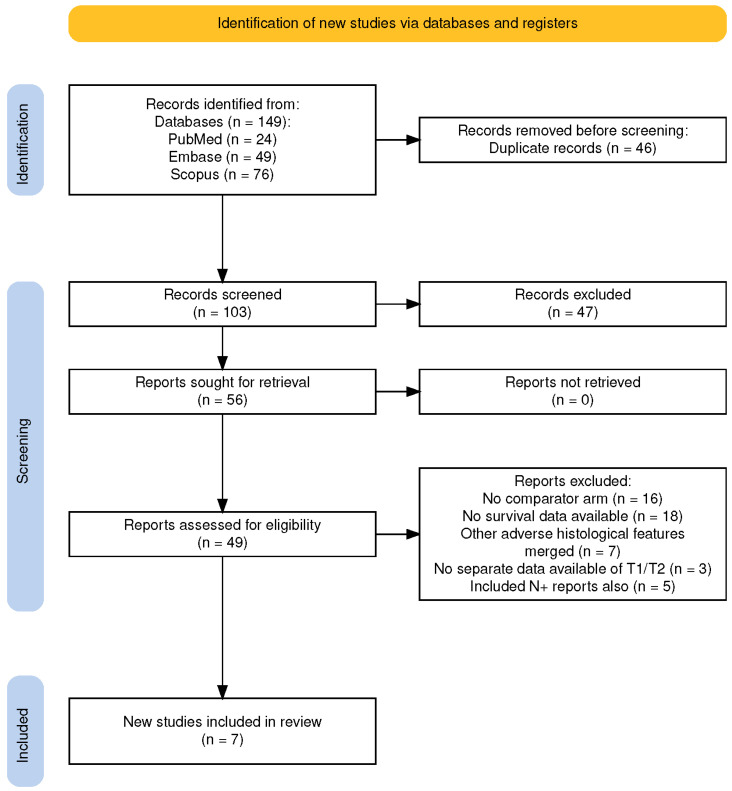
PRISMA flow diagram.

The database search was conducted across PubMed, EMBASE, and Scopus, resulting in 24, 49, and 76 articles, respectively, totaling 149 articles. After removing 46 duplicates, 103 articles remained for title screening. During this phase, 47 articles were excluded for including non-oral-cavity sites, advanced disease stage, or included adjuvant chemoradiotherapy, leaving 56 articles for abstract screening. None of the articles were excluded due to unavailability of the full text. Following abstract and full-text screening, 49 articles were excluded due to reasons such as having no comparator arm (16 articles), no survival data available (18 articles), merging of other adverse histological features (7 articles), lack of separate data for T1/T2 (3 articles), and inclusion of N+ reports (5 articles). Ultimately, seven studies [12,13,14,15,16,17,18] were included in the final review (Figure 1).

**Figure 2:**
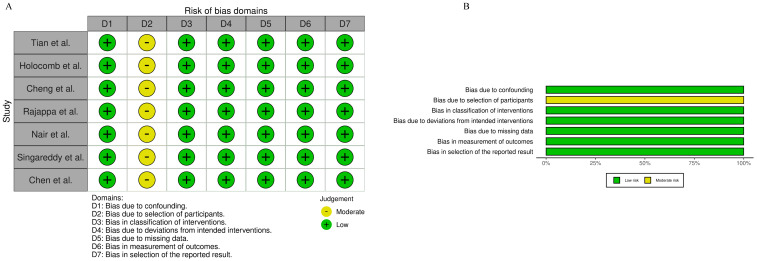
Risk of bias [12,13,14,15,16,17,18]: (**A**) graph; (**B**) summary.

The qualitative data synthesis yielded 522 cases of node-negative early-stage OSCC with PNI from seven studies. Of these, 281 patients received PORT and 248 did not receive PORT. All eligible studies were retrospective cohort studies, and none were randomized controlled trials (Table 1). Based on the risk of bias assessment tool, the included studies had the highest risk of patient selection and confounding variables (Figure 2).

### 3.1. Three-Year OS

**Figure 3:**
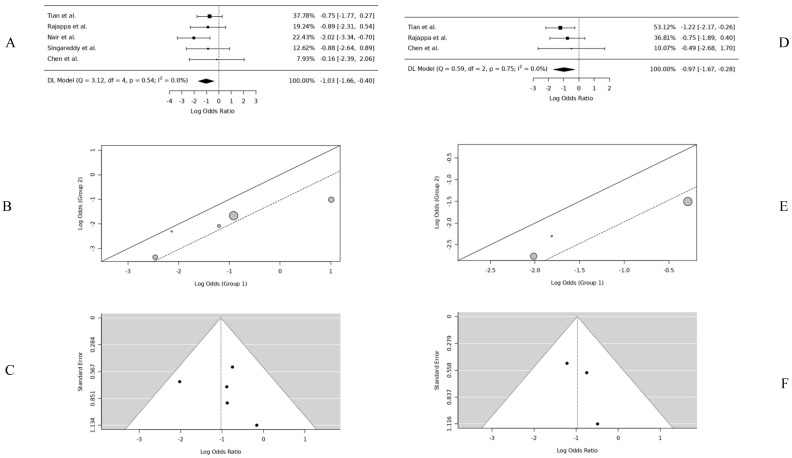
Three-year OS forest plot [12,13,14,15,17] (**A**), three-year OS L’Abbé plot [12,13,14,15,17] (**B**), three-year OS funnel plot [12,13,14,15,17] (**C**), five-year OS forest plot [12,15,17] (**D**), five-year OS L’Abbé plot [12,15,17] (**E**), five-year OS funnel plot [12,15,17] (**F**).

The 3-year OS data were extracted from five studies [12,13,14,15,17] for PORT and no-PORT in T1/T2/N0 with PNI cases for meta-analysis. The pooled 3-year OS was 86.3% for PORT (*n* = 245) and 71.1% for no-PORT (*n* = 185). The pooled data had no significant heterogeneity or publication bias. The logOR was −1.03 [(−1.65, −0.4), DL, REM, CI = 95%], significantly favoring PORT (*p* = 0.0012). *The probability of 3-year OS with PORT was 73.7%* (Table 2) (Figure 3).

### 3.2. Five-Year OS

The 5-year OS data were extracted from three studies [12,15,17]. The pooled 5-year OS was 88.1% for PORT (n = 173) and 77.3% for no-PORT (n = 157). The pooled data had no significant heterogeneity or publication bias. The logOR was −0.97 [(−1.66, −0.27), DL, REM, CI = 95%], significantly favoring PORT (*p* = 0.0061). *The probability of 5-year OS with PORT was 72.6%* (Table 2) (Figure 3).

### 3.3. Three-Year DFS

**Figure 4:**
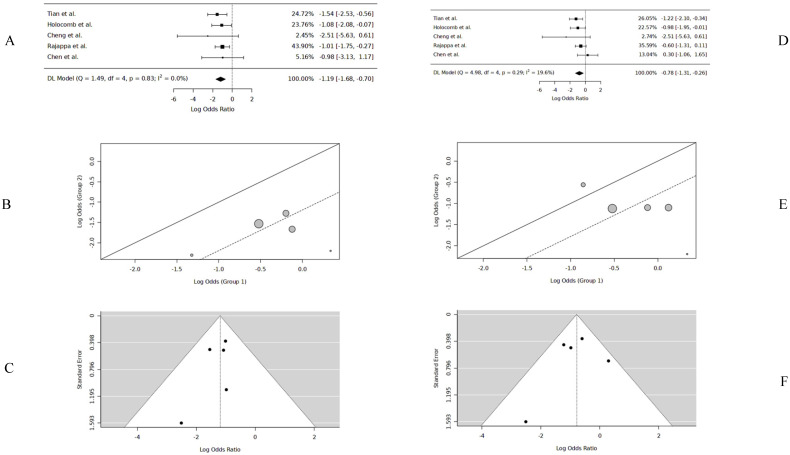
Three-year DFS forest plot [12,15,16,17,18] (**A**), three-year DFS L’Abbé plot [12,15,16,17,18] (**B**), three-year DFS funnel plot [12,15,16,17,18] (**C**), five-year DFS forest plot [12,15,16,17,18] (**D**), five-year DFS L’Abbé plot [12,15,16,17,18] (**E**), five-year DFS funnel plot [12,15,16,17,18] (**F**).

The 3-year DFS data were extracted from five studies [12,15,16,17,18]. The pooled 3-year DFS was 86.3% for PORT (n = 209) and 58.1% for no-PORT (n = 220). The pooled data had no significant heterogeneity or publication bias. The logOR was −1.19 [(−1.68, −0.7), DL, REM, CI = 95%], significantly favoring PORT (*p* < 0.001). *The probability of 3-year DFS with PORT was 76.8%* (Table 2) (Figure 4).

### 3.4. Five-Year DFS

The 5-year DFS data were extracted from five studies [12,15,16,17,18]. The pooled 5-year DFS was 86.3% for PORT (n = 209) and 55% for no-PORT (n = 220). The pooled data had no significant heterogeneity or publication bias. The logOR was −0.78 [(−1.30, −0.25), DL, REM, CI = 95%], significantly favoring PORT (*p* = 0.003). *The probability of 5-year DFS with PORT was 68.7%* (Table 2) (Figure 4).

### 3.5. Three-Year LC

**Figure 5:**
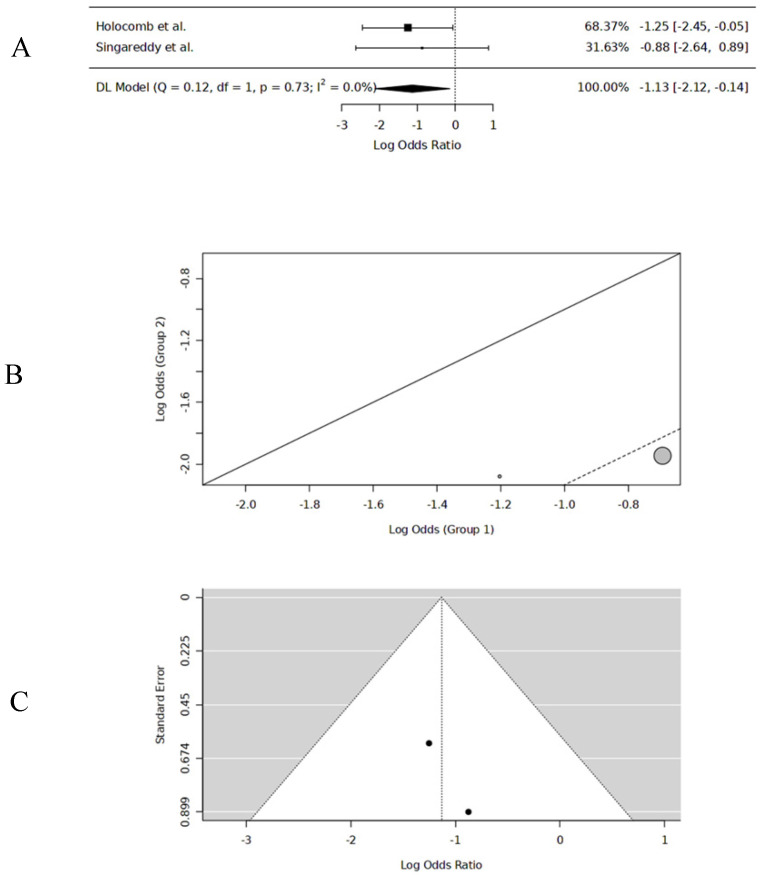
Three-year LC forest plot [13,18] (**A**), three-year LC L’Abbé plot [13,18] (**B**), three-year LC funnel plot (**C**).

The 3-year LC data were extracted from two studies [13,18]. The pooled 3-year LC was 89% for PORT (n = 59) and 72.2% for no-PORT (n = 64). The pooled data had no significant heterogeneity or publication bias. The logOR was −1.13 [(−2.12, −0.14), DL, REM, CI = 95%], significantly favoring PORT (*p* = 0.025). *The probability of 3-year DFS with PORT was 75.7%* (Table 2) (Figure 5).

## 4. Discussion

Managing OSCC poses a significant challenge even for an experienced multidisciplinary team. Approximately 30 to 40% of OSCCs are detected at an early stage, and surgical intervention remains the gold standard [3,4]. In T1/T2N0 oral cancers, PORT is not recommended. Our study specifically examined whether the presence of PNI alone in T1/T2N0 oral cancers justifies the use of PORT in patients who would otherwise be managed with observation alone.

PNI involves the spread of tumor cells along nerves and is influenced by, among other things, the nerve microenvironment [35]. Initially described by Batsakis et al. as tumor involvement in, around, and through nerves [36,37], Liebig et al. later defined PNI as a presence of tumor cells in the epineurium, perineural space, and nerve sheath or encasing the nerve more by than 33% of its circumference [38]. However, these definitions do not distinguish between perineural spread (detection of tumor cells in and around the perineural space) and intraneural spread (tumor cell penetration within the nerves), which can affect prognosis [39]. PNI location (intratumoral or peripheral), PNI density (number of PNI foci per tissue per section), and involved nerve size (major nerves or nerves with small sizes) may also have a prognostic impact. Only in two of the included studies was PNI defined [12,14]. Rajappa et al. used the finding of tumor cells within any of the layers (epineurium, perineurium, or endoneurium) of the nerve sheath or invasion of cancer cells in, around, and through nerves for defining perineural invasion [12]. When the tumor cells were not found within the nerve sheath, perineural invasion was defined as at least a third of the circumference of the nerve being surrounded by tumor cells. Nair et al. considered the tumor PNI positive when, microscopically, at least 33% of the nerve circumference was surrounded by tumor cells [14]. Brandwein-Gensler et al. demonstrated that PNI independently predicts local recurrence and overall survival, regardless of tumor margins [40]. Numerous studies emphasize the benefit of PORT when PNI is present, leading to improved oncological outcomes [12,15,18,21].

In our meta-analysis focusing on T1/T2/N0 cases with PNI, the pooled data from multiple retrospective studies indicated that PORT significantly enhanced outcomes. PORT significantly improved 3-year OS (86.3% vs. 71.1%, *p* = 0.0012) and 5-year OS (88.1% vs. 77.3%, *p* = 0.0061). It also enhanced 3-year DFS (*p* < 0.001), 5-year DFS (*p* = 0.003) and 3-year LC (89% vs. 72.2%, *p* = 0.025). The included studies used the standard dosage of 60 Gy in 30 fractions for PORT, but data on the PORT technique were not available.

The current National Comprehensive Cancer Network (NCCN) guidelines recommend PORT for patients with T1/T2/N0 OSCC who exhibit adverse histological features, including PNI [6]. These recommendations quote the seminal EORTC-22931 study by Bernier et al., which included 87 patients with OSCC. Notably, 33% of the cohort consisted of T1/T2 cases with N2 and N3 staging. This study compared PORT versus postoperative chemoradiotherapy, without an observation arm [41].

It is crucial to highlight that the National Cancer Database (NCDB) does not provide data on PNI, thus limiting large-cohort analyses on the impact of adjuvant therapy in this context [42]. PORT is associated with potential late complications, such as xerostomia, dysphagia, and osteoradionecrosis, and its administration thus warrants thorough consideration [43].

It is important to recognize that PNI encompasses various parameters beyond a simple dichotomous (yes or no) classification, including factors like PNI depth, length, density, and involved nerve thickness. The study by Brandwein-Gensler et al. underscores the fact that PNI greater than one millimeter correlates with poorer outcomes and higher rates of local recurrence [40]. They further stratified the adverse histopathological features into low-, intermediate-, and high-risk categories for early-stage OSCC based on PNI, invasion pattern, and tissue–tumor interface. Notably, only the high-risk group demonstrated improved oncological outcomes with adjuvant RT. It essential to note that the studies referenced by Brandwein-Gensler et al. are over 15 years old, predating significant advancements in radiation therapy techniques and our understanding of tumor biology [40]. Therefore, the reassessment of risk stratification using newer datasets is warranted. Our evolving knowledge of PNI biology, as highlighted by the studies of Aivazian et al. [44] and Hasmat et al. [20], suggests that multifocal PNI may portend worse outcomes compared to unifocal PNI in early-stage OSCC. They also highlight that the occurrence of multifocal PNI in early-stage OSCC is uncommon.

The present meta-analysis exhibits several limitations. The studies often had small sample sizes and retrospective designs confined to single institutions, which may introduce selection bias and hinder generalizability. The decision to administer PORT should therefore be carefully weighed, as it comes with potential toxicity risks, which has not been described in the studies. There was a lack of consensus on the definition of PNI, particularly concerning multifocal PNI and PNI density, which complicates the interpretation and comparison of the study findings. Variability in surgical practices, including differences in resection extent and neck dissection, further obscure outcomes across the studies. Additionally, data specific to different subsites within the oral cavity, depth of PNI invasion, margin status, and other histopathological features were inconsistently reported or analyzed. Details regarding radiation therapy, such as type and planning, were often insufficiently described, limiting the assessment of treatment effects. Furthermore, the studies frequently treated PNI as a dichotomous variable, overlooking potential variations in PNI characteristics that could impact prognosis differently. Addressing these limitations through prospective multicenter studies with standardized methodologies and comprehensive data collection would significantly advance our understanding and management of PNI in OSCC.

## 5. Perspectives and Recommendations

Based on current analyses, the evidence suggests that adjuvant PORT improves oncological outcomes in early-stage OSCCs with PNI. However, it is important to note that this evidence is primarily derived from retrospective studies, which may have some degree of selection bias. Therefore, it is best clinical practice to fully inform patients about the risks and benefits of radiation therapy, including its impact on oncological outcomes, recurrence rates, potential side effects such as mucositis and xerostomia, and associated costs. The decision to proceed with adjuvant therapy should be reached through mutual agreement between the multidisciplinary team and the patient. This decision is also dependent on other adverse features and the patient’s preference. To strengthen the existing evidence, there is a critical need for well-designed, multicentered, randomized controlled trials with robust statistical power and rigorous long-term follow-up. This approach would provide the highest-quality evidence to guide future treatment decisions.

## 6. Main Conclusions

This meta-analysis of retrospective studies suggests that adjuvant PORT for patients with a node-negative early-stage OSCC with PNI tends to improve OS, DFS, and LC.

## Figures and Tables

**Table 1:** Overview of included studies.

Author	Year	Country	Study	OCEBM	Site	Stage	No. of PNI	PNI with PORT	PNI with No-PORT	3y OS PORT (%)	3y OS No-PORT (%)	5y OS PORT (%)	5y OS No-PORT (%)	3y DFS PORT (%)	3y DFS No-PORT (%)	5y DFS PORT (%)	5y DFS No-PORT (%)	3y DSS PORT (%)	3y DSS No-PORT (%)	5y DSS PORT (%)	5y DSS No-PORT (%)	3y LC PORT (%)	3y LC No-PORT (%)	5y LC PORT (%)	5y LC No-PORT (%)
Tian et al. [15]	2024	China	R	3b	Tongue	T1/2N0	89	44	49	84.5	71.6	81.4	58.1	84	52.3	76	47.6								
Holocomb et al. [18]	2023	USA	R	3b	Oral Cavity	T1/2N0	83	32	51					78.5	55.2	74.5	52.2					89	67.5	84.7	63.7
Cheng et al. [16]	2022	Taiwan	R	3b	Oral Cavity	T1/2N0	16	4	12					100	42.3	100	42.3	100	38.6	100	38.6				
Rajappa et al. [12]	2019	India	R	3b	Oral Cavity	T1/2N0	169	118	51	96.6	91.2	93.9	88.7	82.2	62.7	75.6	62.7								
Nair et al. [14]	2018	India	R	3b	Oral Cavity	T1/2N0	60	45	15	72.6	27.3														
Singareddy et al. [13]	2016	India	R	3b	Oral Cavity	T1/2N0	40	27	13	88.9	77											88.9	76.9		
Chen et al. [17]	2013	Taiwan	R	3b	Oral Cavity	T1/2N0	65	11	57	88.9	88.6	88.9	85.2	86.6	78.2	61.1	70.2								
							522	281	248	86.3	71.1	88.1	77.3	86.3	58.1	77.4	55.0	100.0	38.6	100.0	38.6	89.0	72.2	84.7	63.7

**Table 2:** Overview of meta-analysis statistics.

			3y OS	5y OS	3y DFS	5y DFS	3y LC
Number of studies (k)			k = 5	k = 3	k = 5	k = 5	k = 2
Log odds ratios (range)			−1.66 to −0.4	−1.67 to −0.28	−1.68 to −0.7	−1.31 to −0.26	−2.1 to −0.14
Majority of estimates		Direction	Negative	Negative	Negative	Negative	Negative
		% Studies	100%	100%	100%	80%	100%
Estimated average log odds ratio			−1.03	−0.97	−1.79	−0.78	−1.13
Odds ratio			0.36	0.37	0.3	0.45	0.32
Survival probability			73.7%	72.6%	76.8%	68.7%	75.7%
Significance of average outcome	Z score		−3.22	−2.7	−4.77	−2.9	−2.2
	*p* value		0.0012	0.0061	<0.001	0.0034	0.025
Maximum weightage	Study		Tian et al. [15]	Tian et al. [15]	Rajappa et al. [12]	Rajappa et al. [12]	Holocomb et al. [18]
	% weight		37.78%	53.12%	43.9%	35.59%	68.3%
Heterogeneity	Q test		3.12	0.58	1.4	4.97	0.12
	tau^2^		0	0	0	0.071	0
	I^2^		0%	0%	0%	19.63%	0%
Outliers	studies		None	None	None	Chen et al. [17]	None
Influential studies	Cook’s distances		>1	<1	<1	<1	<1
	studies		Nair et al. [14]	None	None	None	None
Funnel plot asymmetry	Rank correlation *p*-value		0.81	1	0.23	1	1
	Egger’s regression test *p*-value		0.71	0.55	0.5	0.59	0.56

## Data Availability

Data are available upon reasonable request from the corresponding author.
